# Ford and Edison in a modern regulatory environment: the first-in-human trial of night-work and artificial light

**DOI:** 10.1186/s12995-017-0154-9

**Published:** 2017-03-16

**Authors:** Thomas C. Erren, David M. Shaw, Ursula Wild, J. Valérie Groß

**Affiliations:** 1 Institute and Policlinic for Occupational Medicine, Environmental Medicine and Prevention Research, University Hospital of Cologne, Cologne, Germany; 2 Institute for Biomedical Ethics, University of Basel, Basel, Switzerland; 3 Department of Health, Ethics and Society, CAPHRI Research Institute, Maastricht University, Maastricht, the Netherlands

**Keywords:** Shift work, Night work, Light, Chronobiology, Chronodisruption, Public health, Thought experiment, Ford, Edison

## Abstract

A thought experiment places Henry Ford and Thomas Alva Edison in a modern regulatory environment. In a utopian occupational world devoid of night-shifts or artificial light, Ford wants to experiment with “working through the night”. To support Ford’s project, Edison offers his patented electric lamps to “turn nights into days”. An ethics committee [EC] does not approve the night-work experiment and Utopia’s Food and Drug Administration [FDA] does not approve the potential medical device as safe for use by humans. According to the EC and FDA, complex effects on circadian biology and thus safety of work and light at night are not understood. The thought experiment conveys that we should pay more attention to possible risks of work and light at chronobiologically unusual times.

There are these two young fish swimming along and they happen to meet an older fish swimming the other way, who nods at them and says, “Morning, boys. How’s the water?”

And the two young fish swim on for a bit, and then eventually one of them looks over at the other and goes,

“What the hell is water?”

-David Foster Wallace [1]

This Is water

## Thought experiment: placing Edison & Ford in a modern regulatory environment

Imagine the industrialist Henry Ford and the inventor Thomas Alva Edison working in our modern regulatory environment. Imagine further a world without work around the clock, with workers clocking off as the sun sets. Imagine finally that Ford contemplates making it possible to work over 24 h rather than 12 h in Utopia. To support this, Edison offers to facilitate work at night with his patented electric lamps.

Utopia’s Unions are immediately on alert. Could the flagship entrepreneurs’ plans jeopardize the daytime working world which people cherish? Disagreeing with such “unnatural” enterprise, Utopia’s Unions ask: “Are work and artificial light at night really safe for humans?” To demonstrate long-term safety, Ford and Edison propose a long-term experiment with individuals working at nighttime exposed to artificial light.

A research proposal is submitted to Utopia’s Ethics Committee [EC]. After all, Ford and Edison argue from their winter retreat in Fort Myers, did changed illumination levels not improve workers’ satisfaction and efficiency at the Hawthorne Works [referred to in [2]]? The EC approaches the patent office [PO] which granted Edison’s patent of an electric lamp. After considering evidence suggesting that light impacts on circadian biology in many ways, the PO specifies that, strictly speaking, electric lamps could be viewed as medical devices.

This led the EC to the following conclusion: The proposed study would experiment with workers’ days and nights. Light and darkness strongly determine day and night in humans. Thus, light is a strong *Zeitgeber* [3, 4]. Light can even qualify as a “functional drug” [5, 6] as it affects circadian biology of humans substantially. Indeed, as voiced by an authoritative researcher, “light affects our circadian rhythms more powerfully than any drug” [7]. Work, usually associated with light, should be considered as a strong *Zeitgeber* as well. In view of evidence that light at night clearly affects circadian biology and since demonstration of long-term safety of work and light at night is lacking, the EC does not approve the long-term experiment.

Thereafter, Utopia’s Food and Drug Administration [FDA] is contacted. The FDA challenges that long-term side-effects of electric lamps, with open questions regarding dose, timing and composition of artificial light, have not been satisfactorily researched. Consequently, light – at chronobiologically unanticipated times, i.e. when individuals tend to sleep [8], cannot necessarily be considered as innocuous and safe. Edison’s repeated assurances [quoted in [7]] that electric light “is in no way harmful to health, nor does it affect the soundness of sleep”, do not persuade the FDA.

As a way out, it is suggested to obtain data from laboratory studies and to submit an Investigational New Drug (IND) application to the FDA’s Center for Drug Evaluation and Research. When this IND application is in effect, Edison may want to instigate clinical trials. Such trials should test whether light as a “functional drug” or electric lamps as “medical devices” are safe in regards to side effects they may cause. If Edison were to conclude – after analyzing the clinical trial data – that there is sufficient evidence on light’s or electric lamps’ safety to meet the FDA’s requirements for marketing approval, Utopia’s FDA envisages signalling to the EC that the use of artificial light as “visually and chronobiologically effective radiant energy for human beings” [9] could be granted for long-term experiments into work at night.

## What does the thought experiment imply?

Back in today’s real world, work and light at night are realities and empirical information that there are short- and medium-term effects associated with respective exposures during individuals’ biological nights [8, 10] is accurate. Table 1 provides examples that both work and light at chronobiologically unusual times can have adverse effects on sleep, the biological night and physiological rhythms of rest and excess.

**Table 1:** Effects of work and light at night

		Work	Light
		Zeitgeber^EthicsCommittee^	Zeitgeber
		Adverse Effects	
Sleep	Sleepiness	Yes [7, 15]	Yes [7]
	Sleep quality	Yes [15]	Yes [7]
	Sleep quantity	Yes [15]	Yes [7]
	Sleep timing	Yes [15]	Yes [7]
	Accidents	Yes [15, 16]	Yes [17]
Biological Night	Restitutional resource [18]	Yes [19]	Yes [20]
Rhythms of Rest and Excess	Physiological rhythms of rest and excess [13]	Yes [13]	Yes [13]

With work and light at night as cornerstones of our lives, the thought experiment conveys “that the most obvious, ubiquitous, important realities are often the ones that are hardest to see and talk about” [1]. Put differently, work and light at night are two of our “most obvious, ubiquitous, important realities” which we consider as natural companions without hesitation.

Disconcertingly, the International Agency for Research on Cancer [IARC] classified shift-work that involves circadian disruption as probably carcinogenic to humans (Group 2A) [11, 12]. Note that the verdict was based on “sufficient evidence in experimental animals for the carcinogenicity of light during the daily dark period (biological night)” and on the fact that “Among the many different patterns of shift-work, those including nightwork are the most disruptive for the circadian clock” [11].

Clearly, the thought experiment does not suggest that we rid ourselves of work and light at night – now that would be a utopian notion! Yet, equally clearly, it may stimulate awareness that we need research to understand what precise risks might be involved when workers in factories and residents in social settings are exposed to *Zeitgeber* information by work and light which conflicts with individuals’ propensities for wake and sleep. We should not ignore that rhythms of rest and excess [13] over nights and days are evolutionary legacies. They provided competitive edges in light–dark cycle-associated environments which species on earth began to anticipate millions of years ago to prepare their bodies for what was coming.

## Conspectus

An IARC Group 1 classification of work at night [=human carcinogen] could be met with denial, skepticism, or disbelief by experts and non-experts alike. Yet, given today’s facts, it could become reality. Note further that links between work and light and “circadian disruption” and cancer may be just the “tip of the iceberg” [14] as a wide spectrum of diseases – including sleep disorders, depression, obesity, diabetes, metabolic syndrome, cardiovascular and neurodegenerative disease – has been implicated in possible causal networks.

Our thought experiment is both fictitious and factual. It conveys that most humans think about work and light at night as much as fish may wonder about water. Scientific facts should change this state-of-affairs. Evolutionarily, neither work nor light at chronobiologically unusual times are “natural” and necessarily benign. We should pay more attention to possible dangers of work and light at night.
